# Nutritional Composition and Estimated Iron and Zinc Bioavailability of Meat Substitutes Available on the Swedish Market

**DOI:** 10.3390/nu14193903

**Published:** 2022-09-21

**Authors:** Inger-Cecilia Mayer Labba, Hannah Steinhausen, Linnéa Almius, Knud Erik Bach Knudsen, Ann-Sofie Sandberg

**Affiliations:** 1 Food and Nutrition Science, Department of Biology and Biological Engineering, Chalmers University of Technology, 412 58 Gothenburg, Sweden; 2 Department of Animal Science, Aarhus University, 8830 Tjele, Denmark

**Keywords:** meat substitutes, meat analogues, plant-based, plant protein, protein shift, phytate, phy:Fe molar ratio, phy:Zn molar ratio, sustainable nutrition, iron, zinc, bioavailability

## Abstract

Transition towards plant-based diets is advocated to reduce the climate footprint. Health implications of a diet composed of meat substitutes are currently unknown, and there are knowledge gaps in their nutritional composition and quality. Samples of available meat substitutes were bought in two convenience stores in the city of Gothenburg, Sweden, and were included in the study. Meat substitutes (*n* = 44) were analyzed for their contents of dietary fiber, fat, iron, zinc, phytate, salt, total phenolics and protein, as well as for their amino acid and fatty acid composition. Bioavailability of iron and zinc was estimated based on the phytate:mineral molar ratio. We found large variations in the nutritional composition of the analyzed meat substitutes. Amino acid profiles seemed to be affected by processing methods. Mycoprotein products were rich in zinc, with a median content of 6.7 mg/100 g, and had very low content of phytate, which suggests mycoprotein as a good source of zinc. Degradability of fungal cell walls might, however, pose as a potential aggravating factor. None of the products could be regarded as a good source of iron due to very high content of phytate (9 to 1151 mg/100 g) and/or low content of iron (0.4 to 4.7 mg/100 g). Phytate:iron molar ratios in products with iron contents >2.1 mg/100 g ranged from 2.5 to 45. Tempeh stood out as a protein source with large potential due to low phytate content (24 mg/100 g) and an iron content (2 mg/100 g) close to the level of a nutrition claim. Producers of the products analyzed in this study appear to use nutritional claims regarding iron that appear not in line with European regulations, since the iron is in a form not available by the body. Meat substitutes analyzed in this study do not contribute to absorbed iron in a relevant manner. Individuals following mainly plant-based diets have to meet their iron needs through other sources. Salt and saturated fat were high in certain products, while other products were more in line with nutritional recommendations. Further investigation of the nutritional and health effects of protein extraction and extrusion is needed. We conclude that nutritional knowledge needs to be implemented in product development of meat substitutes.

## 1. Introduction

A dietary shift from animal products, especially meat, into plant-based diets is rapidly expanding as awareness of climate and health effects associated with excessive red and processed meat consumption [1,2,3] is raising interest in plant-based food patterns [4,5]. In general, a high intake of whole plant foods low in salt, saturated fats and added sugars is recommended as part of a healthy lifestyle [6]. On the contrary, a lower-quality plant-based diet has been negatively associated with metabolic health as the quality of the diet, rather than simply being plant-based, not surprisingly has been shown to be more important in promoting health [7,8,9,10]. Technological progress during recent years appears to be an important driver for the emerging interest in plant-based diet, as technology has made it possible for imitation of meat products using plant components. Plant-based meat analogues are food products simulating the aesthetic and sensory characteristics of traditional meat products and are heavily growing in demand in Western societies [11,12]. The use of refined meat substitutes is becoming increasingly common [13,14,15], with the main reason for choosing plant meat substitutes associations of it being “better for you” and “better for the planet” [16]. Previous work points out that many novel meat substitutes have high contents of salt and saturated fat [17,18,19]. Estell et al. [20] showed that iron content is an important criteria for consumers when choosing meat alternatives, and that there is an expectation that the availability of iron in meat substitutes is comparable to that of red meat. While iron content has been reported as generally high in meat substitutes [19,21], there is reason to question the bioavailability of minerals in meat substitutes, especially those based on protein extracts, as phytate, a potent inhibitor of mineral absorption, is known to accumulate in the protein fraction [22,23,24,25,26]. There are, therefore, substantial knowledge gaps in the nutritional composition and quality of meat substitutes. Although some consumers promote them as sustainable and healthy compared to the meat products they aim to replace [27], little research has critically evaluated the impact of meat alternatives on public health and food systems.

In the present study, we examined the nutritional composition, total phenolic content and levels of mineral-absorption-inhibiting phytate, and estimated the iron and zinc bioavailability of meat substitutes commonly available on the Swedish market. The aim of the study was to investigate if there are nutritional limitations connected to including meat substitutes in the diet.

## 2. Materials and Methods

### 2.1. Collection and Preparation of Samples

Two common and representative supermarkets in central Gothenburg were selected. The stores selected were ICA and Willys, two of Sweden’s largest supermarket chains found across the country. From these two supermarkets, one package of each available meat substitute was bought, with the reservation of not buying the same item twice. Each sample (*n* = 44) was freeze-dried, ground using a coffee grinder and stored at −18 °C until further analysis. Samples were collected during August 2021.

### 2.2. Mineral Analyses

The contents of iron and zinc were determined in duplicate by atomic absorption spectrometry (200 Series AA Systems; Agilent). Prior to analysis, samples were digested using a microwave (Milestone microwave laboratory system; EthosPlus, Sorisole, Italy) at acidic conditions, as previously described by Fredrikson et al. [28]. Calibration was performed using commercial standards with a concentration range of 0.125–5.0 mg/L. The limit of detection (LOD) of the method is 0.1 mg/L.

### 2.3. Phytate Analysis

Phytate was analyzed as inositol hexaphosphate (InsP_6_) by high-performance ion chromatography (HPIC) according to Carlsson et al. [29]. A sample of freeze-dried and ground meat substitute (0.5 g) was extracted in duplicate with 10 mL of 0.5 mol/L HCl for three hours using a laboratory shaker (Heidolph Reax 2; Heidolph Instruments, GmbH, Schwabach, Germany). Then, 1 mL was removed, centrifuged and filtered to remove fat before transferring the extract to an HPLC vial. The chromatography setup consisted of an HPLC pump (model PU-400oi; Jasco Inc., Easton, MD, USA) for the eluent and an RHPLC pump (model PU-4180; Jasco, Oklahoma City, OK, USA) equipped with a PA-100 guard column and a CarboPac PA-100 column. InsP_6_ was eluted with an isocratic eluent of 80% HCl (1 mol/L) and 20% MilliQ water at 0.8 mL/min, subjected to a post-column reaction with ferrous nitrate, and detected at 290 nm in a UV–visible HPLC detector (UV-4075; Jasco, Oklahoma City, OK, USA). Columns were run under ambient temperature. Each sample had a run time of 7 min, and the InsP_6_ concentration was calculated using external standards covering the range of 0.1–0.6 μmol/mL.

### 2.4. Estimation of Iron and Zinc Bioavailability

The molar ratios of phy:Fe and phy:Zn were calculated to estimate the bioavailability of iron and zinc, respectively, and to give an indication of the inhibitory effects of phytates on these minerals. The molar ratio of phy:mineral is commonly used as a simple method to estimate the bioavailability of Ca, Fe and Zn in the presence of phytate. Since inhibition due to phytate on both non-heme iron and zinc absorption is dose-dependent in human subjects, a low phy:mineral molar ratio correspond to high theoretical bioavailability [30,31,32]. According to the European Food Safety Authority (EFSA), a phy:Zn molar ratio below five corresponds to high absorption efficiency, 5–15 is moderate, and above 15 is low bioavailability [33]. For iron, FAO/INFOODS/IZINCG recommends using the phy:Fe molar ratios presented as dietary reference values suggested by Hurrell and Egli [34,35]. According to these recommendations, the phy:Fe molar ratio should be below 1, or preferably below 0.4, to significantly improve non-heme iron absorption in plant-based meals with no iron absorption stimulating factors. In meals simultaneously containing the so called “meat factor” (muscle tissue from meat, fish or poultry) and ascorbic acid, the phy:Fe molar ratio should not exceed 6. In this paper, we have used these recommended thresholds to evaluate the bioavailability of iron and zinc in meat substitutes. A molecular mass of 660.3 g/mol for phytate was used for the calculations.

### 2.5. Determination of Total Phenolic Content

The total phenolic content (TPC) was determined by the Folin–Ciocalteu method based on the technique of Howard et al. [36] with some modifications.

Duplicate samples of dried and ground meat substitute (0.8 g) were mixed thoroughly with 5 mL of methanol extraction solution (1% trifluoracetic acid in MeOH:H_2_O, 70:30) and then sonicated for 5 min (Branson Ultrasonics Corporation, Danbury, CT, USA). The mixture was vortexed and sonicated again for 5 min, incubated in a shaking water bath (60 °C, 100 rpm) for 30 min and then cooled on ice for 10 min. The extracts were vortexed and centrifuged at 5000× *g* for 5 min at 4 °C. The supernatant was collected, and the pellet was re-dissolved in 5 mL of methanol extraction solution, sonicated as described above, and centrifuged (5000× *g* for 5 min at 4 °C). The second supernatant was added to the previously collected supernatant and stored at −20 °C until analysis. Before use, the extracts were centrifuged at 5000× *g* for 5 min.

For the TPC analysis, Folin–Ciocalteu reagent was added, and the extracts were analyzed spectrophotometrically against a standard curve of gallic acid, measuring the absorbance at 765 nm using a Safire 2 plate reader (Tecan Group Ltd., Männedorf, Switzerland) with the Magellan software. The results for TPC are presented as gallic acid equivalent (GAE) per 100 g of product. LOD of the method is 0.01 mg/mL.

### 2.6. Analysis of Total Fat and Fatty Acid Composition

Extraction of fat was done according to Bligh et al. [37] with slight modifications. In duplicates, 0.2 g of freeze-dried and ground meat substitute was added to 5 mL of chloroform:methanol (2:1) (Sigma Aldrich (St. Louis, MO, USA) and Fischer Scientific (Waltham, MA, USA)) in a glass vial, incubated at room temperature in a rotating shaker (Heidolph Reax 2; Heidolph Instruments GmbH, Schwabach, Germany) for 30 min and then sonicated (Branson ultrasonic bath model 8510-DTH, Sigma Aldrich St. Louis, MO, USA) in a water bath for 5 min. The incubation and sonication steps were repeated twice. To stop the reaction, 1.5 mL of Milli-Q was added to the vial, which was then centrifuged for 2 min at 4000× *g*. The chloroform layer was collected into a new glass vial, and 4 mL of pure chloroform was added into the previously centrifuged tube to collect any remaining fat. After vortexing and centrifugation at 4000× *g*, chloroform was again removed and pooled. The chloroform:fat solution was then evaporated under nitrogen until the vial maintained a stable weight. Fatty acids were analyzed by direct trans-esterification. Fatty acid methyl esters (FAME) were quantified using GC-MS. Analysis was done according to the method described by Lepage et al. [38] and modified by Cavonius et al. [39]. Peak processing was done in MassHunter Quantitative Analysis Software v2008 (Agilent, Santa Clara, CA, USA).

### 2.7. Determination of Salt Content

As meat substitutes have been criticized for generally high salt content, we analyzed the content of salt in the meat substitutes. An accredited method based on pressure digestion, SS-EN 13805:2015, was used and carried out by Eurofins, Lidköping, Sweden.

### 2.8. Determination of Protein Content

Total nitrogen was determined by the Kjeldahl method and was performed by Eurofins, Lidköping, Sweden. Total protein content was calculated using a nitrogen-to-protein conversion factor of 5.4 for products based primarily on legumes, 5.8 for primarily cereal-based products and 5.85 for primarily cheese-based products [40].

### 2.9. Analysis of Amino Acid Profiles

Amino acid analysis was carried out using a modified version of the method previously described by Özcan et al. [41]. A total of 8 mL of 6 mol/L HCl was added to 100 mg of dried and ground sample, and the mixture was hydrolyzed for 24 h at 110 °C. After hydrolysis, the volume was adjusted to 10 mL using Milli-Q water, and an aliquot of 2 μL was injected into the LC-MS system: an Agilent 1260 HPLC with a Phenomenex column (C18 (2) 250 μm × 4.6 μm × 3 μm), coupled to an Agilent 6120 Quadrupole in the SIM-positive mode (Agilent Inc., Santa Clara, CA, USA). The composition of mobile phase A was 3% MeOH, 0.2% formic acid and 0.01% acetic acid (HAc), and mobile phase B was 50% MeOH with 0.2% formic acid and 0.1% HAc. The initial gradient, held for the first 8 min, contained 94% A and 6% B, which was gradually changed until it reached 80% A and 20% B after 20 min. This gradient was held for a run time of 27 min, then gradually altered until it reached 94% A and 6% B at a run time of 28 min, and then held again for a total run time of 40 min. Twenty-four amino acids diluted in 0.2 mol/L HAc in the concentration range of 1–20 mg/L were used to derive the standard curve. Due to the use of acidic hydrolysis, tryptophan could not be quantified. LOD of the method is 0.025 µmol/mL.

### 2.10. Analysis of Dietary Fiber

Analysis of total, soluble and insoluble dietary fiber was performed using a modification of the enzymatic–chemical–gravimetric Englyst and Uppsala methods [42] at Aarhus University, Denmark, briefly, consisting of the following steps. In duplicates, starch was degraded by thermostable α-amylase, and amyloglucosidase and soluble dietary fiber components precipitated or not with 80% (*v*/*v*) ethanol. The polysaccharides in the starch-free residue were swelled with 12 mol/L H_2_SO_4_ and were hydrolyzed to monosaccharides by 2 mol/L H_2_SO_4_, with lignin (Klason) determined as the acid-insoluble residue. The sugars in the acid hydrolysate were reduced to alcohols and acetylated to alditol acetate, and their concentration was determined by gas–liquid chromatography for neutral sugars and by colorimetry for uronic acids. Total dietary fiber was the sum of neutral and acidic sugar residues in precipitated starch-free residue plus Klason lignin. Insoluble dietary fiber was the sum of neutral and acidic sugar residues plus Klason lignin in the non-precipitated starch-free residue. Soluble dietary fiber was the difference between total dietary fiber and insoluble dietary fiber. Average standard deviations were 0.16% (total dietary fiber), 0.18% (insoluble fraction) and 0.28% (soluble fraction).

### 2.11. Statistics

For descriptive purposes, data are presented as medians and interquartile ranges and presented graphically using boxplots. Since data were non-normally distributed, non-parametric statistical methods were used. Differences in nutrient levels between groups were analyzed via Kruskal–Wallis test, with significant results being followed by pairwise comparisons using Mann–Whitney U test. A *p*-value < 0.05 was considered significant. Statistical analyses were performed using IBM SPSS Statistics v27.0 (IBM, New York, NY, USA).

## 3. Results and Discussion

In total, 44 different meat substitutes were collected and analyzed (Table 1). One additional sample of tempeh was added to the analysis of phytate; this sample is only discussed in the phytate section. Extracted soy protein was the predominant source of protein among the samples analyzed; in total, 17 (39%) contained extracted soy protein. Extracted pea protein was used in 14 (32%) of the products. Results are presented as product weight. Calculations on contribution to daily intake are made on the assumption that one meal is composed of 150 g meat substitute as the protein source.

### 3.1. Content of Iron and Zinc

Soy sausage 1 was excluded from the analysis of iron since it contained the colorant iron oxide, which contaminated the analysis. Analysis using Kruskal-Wallis showed significant differences depending on the main protein source for both iron content (*p* = 0.009) and zinc content (*p* = 0.001).

**Figure 1:**
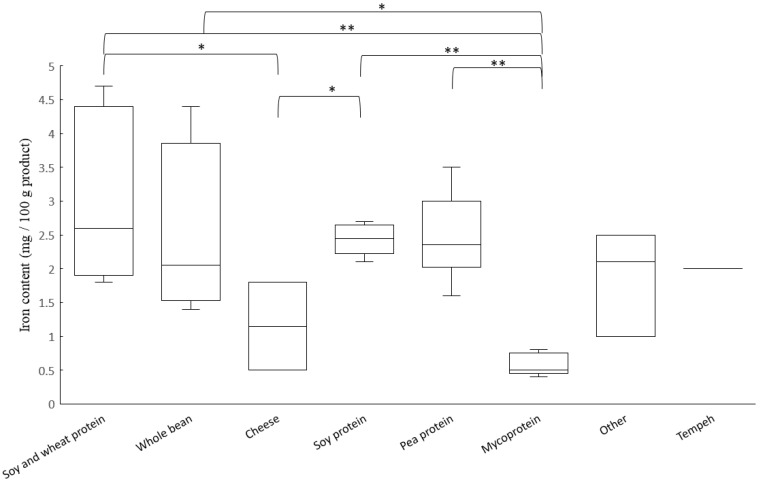
Boxplot of iron content in analyzed meat substitutes, sorted and analyzed according to main source of protein. Line inside the box shows the median value, top of each box is the 75th percentile, and bottom of each box is 25th percentile. Whiskers are maximum and minimum values; small circles are outliers. A *p*-value < 0.05 is illustrated with * and a *p*-value < 0.01 with **.

The iron content varied between 0.4 mg/100 g in the Mycoprotein bites, to 4.6 mg/100 g in the Soy and wheat schnitzel, which was a product fortified with iron. We found large variations in iron content of the analyzed meat substitutes (Figure 1). Products based on mycoprotein had the lowest iron content among categories (median 0.5 mg/100 g). Out of the 44 products, five were fortified with iron (Soy and wheat bacon, Soy and wheat schnitzel, Cheese patties 1, Soy and wheat balls 3 and Chickpea falafel 2). In accordance with our findings, content of iron in meat substitutes based on pea protein has previously been reported as high, while mycoprotein products have been reported to contain a low amount of iron [21,43].

**Figure 2:**
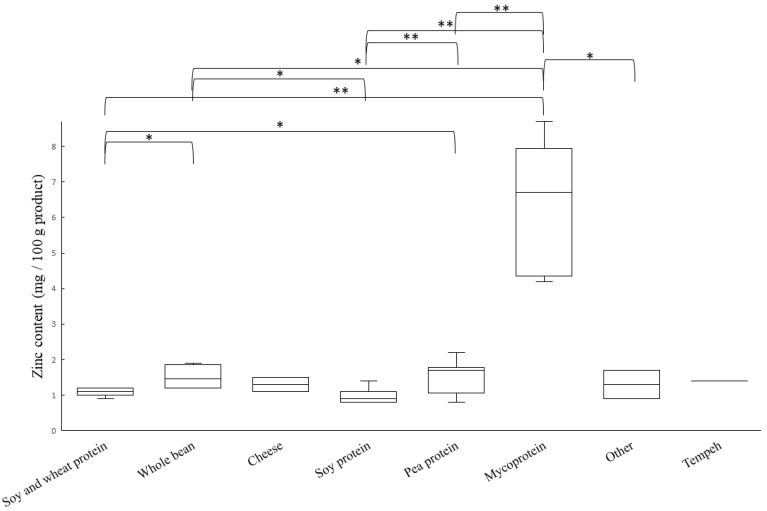
Boxplot of zinc content in meat substitutes, sorted and analyzed according to main source of protein. Line inside the box shows the median value, top of each box is the 75th percentile, and bottom of each box is 25th percentile. Whiskers are maximum and minimum values. A *p*-value < 0.05 is illustrated with * and a *p*-value < 0.01 with **.

The zinc content of meat substitutes (Figure 2), excluding the category Mycoprotein, was in the range of 0.8 mg/100 g (Pea sausage 1) to 2.2 mg/100 g (Pea burger 1), which is considered low. Products based on mycoprotein, however, had a high content of zinc compared to the other meat substitutes, which is consistent with previously reported data from Derbyshire and Ayoob [44]. The mean zinc content of mycoprotein products was 6.7 mg/100 g and was in the range of 4.2 mg/100 g (Mycoprotein burger) to 8.7 mg/100 g (Mycoprotein bites). The contribution of zinc from the meat substitutes analyzed in this study varies to a high degree depending on the product. From a meal containing 150 g of Mycoprotein bites, the meat substitute would contribute 13.1 mg of zinc, which corresponds to 186% of the recommended daily intake (RDI) for women of fertile ages and 145% of the RDI for adult males. None of the products from categories other than Mycoprotein can be considered to contribute to the intake of zinc. This is an important finding, as zinc is identified as a risk nutrient in a mainly plant-based diet [45].

### 3.2. Phytate Content

**Figure 3:**
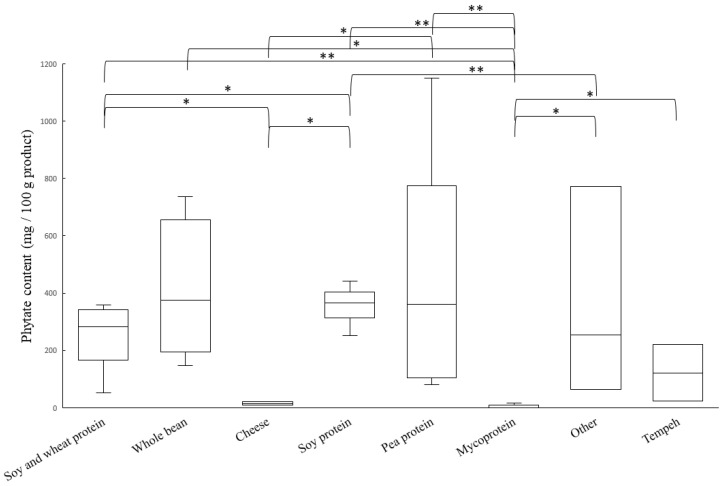
Boxplot of phytate content. Line inside the box shows the median value, top of each box is the 75th percentile, and bottom of each box is 25th percentile. Whiskers are maximum and minimum values. A *p*-value < 0.05 is illustrated with * and a *p*-value < 0.01 with **.

There were large variations in the content of phytate, analyzed as inositol hexaphosphate (InsP6), among samples (Figure 3). Analysis using Kruskal–Wallis showed significant differences in phytate content depending on the main protein source (*p* = 0.039). The lowest content of phytate was found in products based on mycoprotein (<0.01 g/100 g), cheese (15 g/100 g) and one of the tempeh products (24 g/100 g). Fermentation by traditional tempeh starter cultures is known to reduce phytate content significantly under optimal conditions [46,47]. In this study, two tempeh products were analyzed for phytate content: Tempeh bites and Tempeh burgers. Both tempeh products were based on whole peas, which can be assumed to have contained a naturally high phytate content prior to fermentation. To produce tempeh, the raw material, which traditionally is cooked soybean, is fermented with *Rhizopus oligosporus. R. oligosporus* is a fungus that has the ability to produce the phytate-degrading enzyme phytase [46]. The conditions used during fermentation highly affect the reduction of phytate content since the microorganisms and enzymes are dependent on optimal pH, temperature, availability of oxygen and other nutrients, fermentation time as well as the starter culture used [46]. Phytate contents in the tempeh products analyzed in this study were 24 mg/100 g (Tempeh burger) and 220 mg/100 g (Tempeh bites).

Phytate content of 24 mg/100 g can be regarded as low enough to grant adequate absorption of non-heme iron present in the meal, while phytate content of 220 mg/100 g can be expected to have a negative effect on non-heme iron absorption from the meal [31]. Since we only analyzed phytate content in two tempeh products in this study, we cannot draw any general conclusions on phytate content of tempeh apart from highlighting the large potential for fermentation with phytase-producing strains as a method for increased bioavailability of nutrients such as non-heme iron and zinc.

Phytate content in the groups Soy and wheat protein, Whole bean, Soy protein, Pea protein and Other were generally very high. Extraction of legume protein has been shown to accumulate phytate [48], which might explain the very high levels of phytate in most legume-protein products. In this study, the protein extraction technique for each product was not known, nor was the exact formulation or plant cultivar of each product. Since both cultivar [49] and extraction technique [50,51] affect the phytate content of a protein extract, it is of high importance to include nutritional aspects during these steps in production.

### 3.3. Estimated Bioavailability of Iron and Zinc

**Figure 4:**
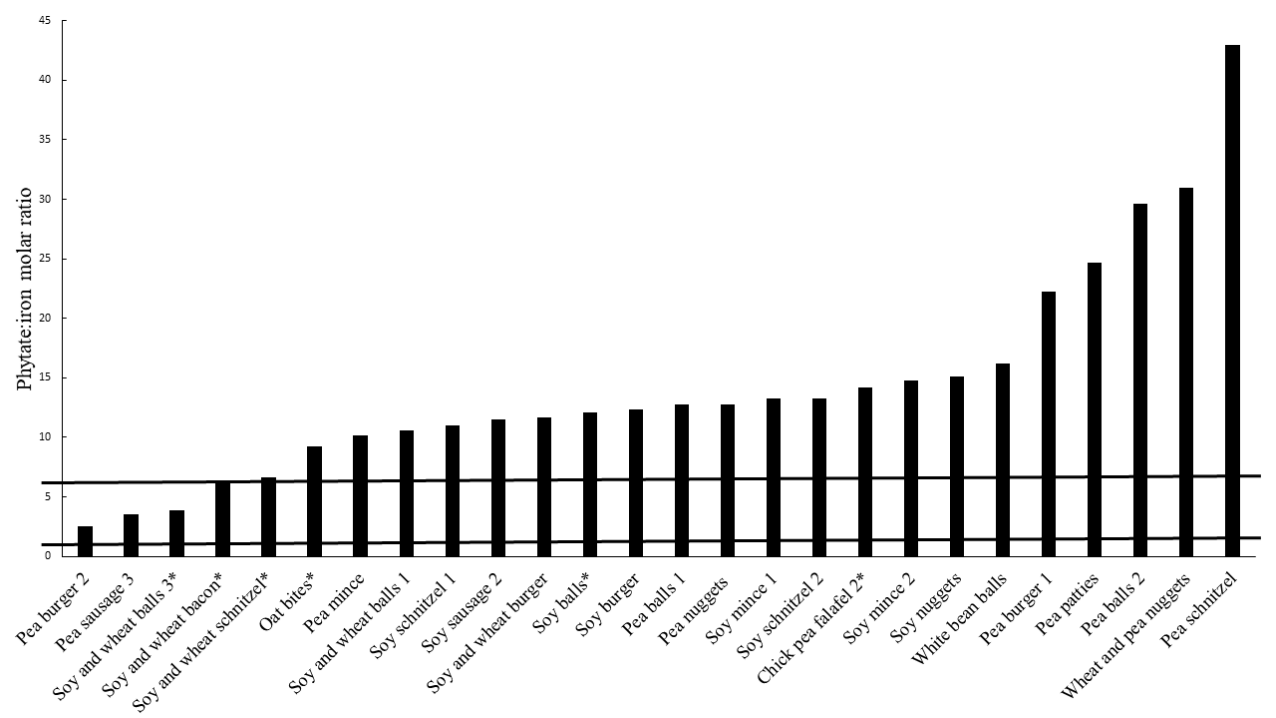
Molar ratio of phytate:iron in meat substitutes with an iron content ≥2.1 mg/100 g. The horizontal lines correspond to a phytate:iron molar ratio of 1 and 6 respectively: * product had a nutrition claim of iron.

Out of the 44 meat substitutes analyzed, 26 (59%) had iron contents ≥2.1 mg/100 g, which is the lower limit for a nutrition claim of iron according to EU regulations [52]. Molar ratios of phytate to iron (phy:Fe), which is an indicator of iron bioavailability, was high among meat substitutes with iron contents ≥2.1 mg/100 g (Figure 4).

A phy:Fe molar ratio of 6 has been suggested as the maximum level at which it is possible to counteract the inhibitory effect of phytate on iron absorption with simultaneous addition of the enhancing factors vitamin C and meat, whereas a phy:Fe molar ratio of maximum 1 [31], or preferably 0.4, has been suggested as limits for adequate bioavailability of iron without stimulating factors [34]. Only three of the products with iron contents of ≥2.1 mg/100 g had a phy:Fe molar ratio ≤6 (Pea burger 2, Pea sausage 3 and Soy and wheat balls 3), and none of the products had a ratio below the limits of 1 or 0.4. These results indicate that the meat substitutes analyzed in this study have very low bioavailability of iron, and that it will be difficult to meet iron needs with a diet mainly consisting of these products as iron sources.

In total, six products had a nutrition claim of iron (Cheese patty 1, Chickpea falafel 2, Oat and bean bites, Soy wheat bacon, Soy wheat balls 3, Soy wheat schnitzel and Soy balls). Cheese patty 1 had an iron content below 2.1 mg/100 g (1.8 mg/100 g) and is hence not presented in Figure 4. The phy:Fe molar ratios of the meat substitutes with a nutrition claim of iron and an iron content of ≥2.1 mg/100 g were 14.2 (Chickpea falafel 2), 9.2 (Oat and bean bites), 6.1 (Soy and wheat bacon), 3.9 (Soy and wheat balls 3), 6.6 (Soy and wheat schnitzel) and 12.1 (Soy balls). Hence, only one of the products, Soy and wheat balls 3, was below the suggested maximum phy:Fe molar ratio for which it is possible to counteract the negative effects of phytate on iron absorption with stimulating factors [34]. The product itself did not contain any enhancing factors for iron absorption.

As mentioned, none of the products were below a molar ratio of 1 or 0.4, which has been suggested as the maximum phy:Fe ratios for adequate absorption of iron without stimulating factors. Apart from a minimum content of a nutrient, the condition “the nutrient for which the claim is made is in a form that is available to be used by the body” has to be fulfilled for a permitted nutrition claim, as stated by the EU regulations on nutrition claims [53]. Hence, a nutrition claim of iron used for a product with a high phy:Fe molar ratio can be argued as not permitted. Moreover, such a claim can also be seen as misleading and negative for consumers aiming to substitute meat, which has a high bioavailability of iron, since it is not possible for the consumer to evaluate the nutritional contribution of such a product.

Poor absorption of dietary iron can cause iron deficiency and iron deficiency anemia, especially among risk groups. Across Western countries, the prevalence of iron deficiency in women of fertile ages has been estimated at 10–30% [54,55,56,57]. At the same time, women across Western societies are twice as likely as men to follow a plant-based diet [58], which stresses the urgent need to improve the nutritional quality of products aimed at substituting meat. In a recent paper, we showed that non-heme iron absorption from a meal with texturized fava bean meal was as low as 4%, compared with 22% from a beef protein meal, in healthy females [59].

### 3.4. Estimated Bioavailability of Zinc

Mycoprotein products were the only meat substitutes analyzed in this study with zinc content above 2.25 mg/100 g, which is the lower limit for a nutrition claim of zinc [52]. Each of the five mycoprotein products included in this study had low contents of phytate, which resulted in very low phy:Zn molar ratios below 0.5. None of the products had a nutrition claim of zinc. According to EFSA, a phy:Zn molar ratio below 5 corresponds to high absorption efficiency, 5–15 is moderate, and >15 is low bioavailability [33]. Hence, mycoprotein products in this study can be regarded as having high bioavailability of zinc as they contained no known inhibitors of zinc absorption. Since zinc is one of the risk nutrients identified in a plant-based diet, this is an important finding. However, nutritional aspects should be incorporated in the development of mycoprotein products, as the nutritional composition of mycoprotein is dependent on species used as well as the composition of the substrate and conditions during biomass growth [60,61]. Digestibility of the cell walls of filamentous fungi is another topic that needs investigation to ensure nutrient bioavailability from mycoprotein products, as cell wall composition varies between species [62].

### 3.5. Total Phenolic Content

The TPC varied in the analyzed meat substitutes between 5.5 GAE/100 g in Pea sausage 3, to 41.0 GAE/100 g in White bean balls (Table 2). While there were large variations within each group, except for products based on mycoprotein, median TPC content was quite constant for products based on protein extracts. The categories Whole bean and Tempeh had the highest median values; however they were not significantly different compared to other categories. Statistical analysis using Kruskal–Wallis showed no significant differences in TPC content between groups sorted according to main protein source.

Products based on whole bean, followed by pea protein products, showed the largest variations of TPC. This might be explained by different formulations, processing techniques and/or cooking conditions. Previous studies have reported a decrease in total phenolic content after extrusion of legumes, with the size of the decrease depending on the conditions during extrusion [63,64,65]. While a reduction in phenolic content might be argued as a negative since phenolic compounds are known to have antioxidant properties, a low level of iron-binding polyphenols in a meal is beneficial for iron bioavailability. Further investigation of specific iron-binding polyphenols of meat substitutes is needed to draw conclusions on potential effects on iron absorption.

### 3.6. Total Fat and Fatty Acid Composition

Since fatty acid composition varies based on the added fat rather than the source of protein, results on fat analyses are sorted into type of added fat as stated on the product package, and not according to protein source. Information on added fat in analyzed meat substitutes can be found in Supplementary Materials Table S1.

We found large variances in the total fat content and fatty acid composition of meat substitutes analyzed in this study. Contents of total fat, monounsaturated fatty acids (MUFA), polyunsaturated fatty acids (PUFA) and essential fatty acids are found in Table 3. Analysis using Kruskal–Wallis showed significant differences depending on fat type in variables total fat (*p* = 0.002), MUFA (*p* = 0.01), PUFA (*p* = 0.03) and essential fatty acids (*n* = 0.03). No significant difference (*p* > 0.05) could be found in variables omega-3 fatty acids and saturated fatty acids).

Total fat varied between 2.0 g/100 g (mycoprotein bites, no added fat) to 21.99 g/100 g (Pea schnitzel, added rapeseed oil). Content of MUFA was in the range of 0.2 (Mycoprotein filet) to 10.79 (Pea schnitzel, added rapeseed oil), and PUFA content varied between 0.8 g/100 g (Mycoprotein filet) to 10.8 g/100 g (Soy schnitzel 2). Essential fatty acid content (ALA and LA) varied between 0.8 g/100 g (Mycoprotein filets, no added fat) to 10.8 g/100 g in Soy schnitzel 2 (added sunflower oil).

**Figure 5:**
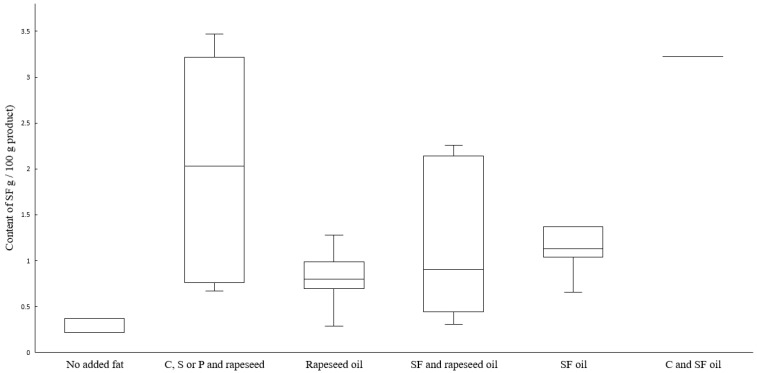
Boxplot of the saturated fatty acid content, sorted and analyzed in meat substitutes based on added fat, as stated on the package. Abbreviations: C, coconut oil; S, shea butter; P, palm oil; SF, sunflower oil. Line inside the box shows the median value, top of each box is the 75th percentile, and bottom of each box is 25th percentile. Whiskers are maximum and minimum values.

**Figure 6:**
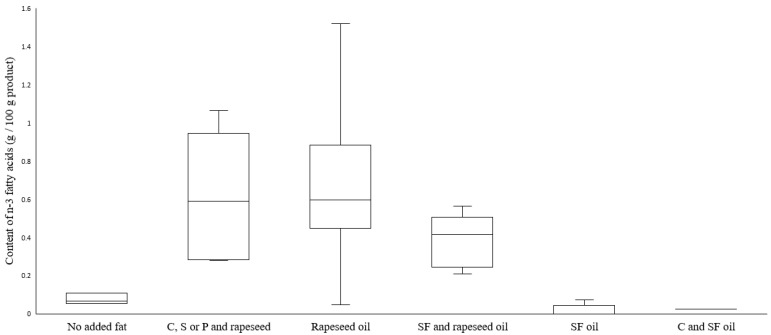
Boxplot of the omega-3 (n-3) fatty acid content, sorted and analyzed in meat substitutes based on added fat, as stated on the package. The only n-3 fatty acid present in the meat substitutes was alpha-linolenic acid (ALA). Abbreviations: C, coconut oil; S, shea butter; P, palm oil; SF, sunflower oil. Line inside the box shows the median value, top of each box is the 75th percentile, and bottom of each box is 25th percentile. Whiskers are maximum and minimum values.

As expected, products with added shea butter or coconut or palm oil had the highest content of saturated fat (Figure 5). Saturated fatty acid content was in the range between 0.2 g/100 g (Mycoprotein filet, no added fat) to 3.5 g/100 g in Pea burger 1 (added shea butter and coconut and rapeseed oil). The omega-3 (n-3) fatty acid content (Figure 6) varied from <0.1 g/100 g in products with added sunflower oil, sunflower and coconut oil, and the two products Mycoprotein mince and Mycoprotein filet that contained no added fat, up to 1.5 g/100 g in Pea schnitzel (added rapeseed oil).

### 3.7. Salt Content

The median content of salt in the analyzed meat substitutes was 1.3 g/100 g and varied between 0.1 g/100 g in the mycoprotein mince, to 2.4 g/100 g in the pea schnitzel (Table 4). Mycoprotein products had a significantly lower content of 0.67 g/100 g salt compared to other meat substitutes in this study, with mycoprotein schnitzel having the highest salt content (1.02 g/100 g). The highest salt content among all samples was found in pea protein, with an average of 1.6 g/100 g (variation between 0.8–2.37 g/100 g).

Since there were large variations in the salt content of the analyzed meat substitutes, the contribution of salt in the diet from meat substitutes varies to a high degree depending on the product. The contribution of salt from 150 g of an average mycoprotein product was 1.0 g (16.7% of maximum intake), which is well within the limits of recommended salt intake according to the Nordic Nutrition Recommendations (NNR) [66], whereas a meal containing 150 g of pea schnitzel contributes 3.6 g of salt. This corresponds to 59.3% of the recommended maximum daily intake of salt based on a limit of 6 g of salt per day [66], which can be considered a substantial contribution.

### 3.8. Protein Content

The protein content varied between 5.5 g/100 g in Pea sausage 1, to 23.8 g/100 g in Oat and bean bites (Table 4). While large variances were seen among samples, median protein content was similar between categories of meat substitutes. No significant difference was found between categories sorted and analyzed based on protein source. The daily protein need of a woman aged 31–64 with a caloric need of 2100 kcal per day is estimated at 53–106 g according to NNR [67]. For an individual belonging to this population group, a meal with 150 g of Pea sausage 1, would contribute 8.3 g of protein (8–16% of the daily protein need), while 150 g of Oat and bean bites would contribute 35.7 g of protein (34–67% of the daily protein need). While contribution of dietary protein is affected by the type of meat substitute consumed, protein deficiency is not a topic of concern among Western populations. The digestibility of protein subjected to different protein extraction and extrusion methods should, however, be investigated further.

### 3.9. Amino Acid Profiles

Proteins from legumes are, in general, low in the indispensable amino acid methionine and high in leucine and lysine. On the contrary, most cereals contain low levels of lysine and high levels of methionine, which make these two groups of plant foods complementary [68]. Therefore, a product containing both cereal and legume protein would be assumed to have a more balanced amino acid profile. However, amino acid composition of products in the category Soy and wheat protein did not reflect this, as they had content of the indispensable amino acid methionine as low as that of the other legume protein categories (Table 5). Previous studies have shown that extrusion cooking affects the amino acid composition, as the measurable levels of certain amino acids decrease during extrusion, with the size of the effect and the amino acids affected dependent on the extrusion conditions [69]. Extruded proteins are hence more complex to evaluate with regards to their amino acid composition, compared with the raw material.

### 3.10. Dietary Fiber

Total dietary fiber varied between 4.4% in the product Wheat fish sticks (category “Other”), to 21.5 in Tempeh burger (Table 6). The insoluble fiber fraction varied between 4.1% in Wheat and pea nuggets (category “Other”) to 15.4 in Tempeh burger. Tempeh burger also had the highest content of soluble fiber with 6.1%; the lowest soluble fiber content was found in Mycoprotein schnitzel (0.7%). Dietary fiber might have beneficial effects in protecting against certain forms of cancer, reducing blood pressure and exerting an anti-inflammatory effect in the digestive tract [70,71]. Certain dietary fibers have also been attributed with a mineral-binding capacity, which might lead to inhibitory effects on mineral absorption [72,73,74]. However, human studies investigating a potential effect of dietary fiber on mineral absorption are scarce and have failed to confirm a negative effect of insoluble and soluble fibers on mineral absorption [75,76]. From a meal containing 150 g of Wheat fish sticks, the contribution to the daily dietary fiber intake would be 3.5 g. A meal with 150 g of Tempeh burger would contribute 9.5 g of dietary fiber. According to NNR, adults are recommended to have a daily dietary fiber intake of 25–35 g [66]. Hence, the contribution from the Wheat fish sticks meal corresponds to 10–14%, and the Tempeh burger meal 27–38% of the recommended daily fiber intake.

Hence, switching to plant-based meat substitutes, especially high-fiber alternatives, could help with reaching recommended intake levels.

Our results reveal large variations in the nutritional composition and quality of meat substitutes on the Swedish market, which is in line with the nutritional composition reported by Bryngelsson et al. [19] based on product labelling. Salt content and fat composition are mainly results of the product formula rather than the inherent composition of plant materials used. Certain products contained a high content of saturated fat and salt, and there was high variation. Amino acid composition of extruded protein products was probably affected by the processing method, as extrusion is known to degrade or convert certain amino acids. Protein content in composite products, as most meat substitutes in this study were, is dependent on the formulation of the product. Covering dietary protein needs for adults with meat substitutes is considered feasible.

Contents of iron, phytate and total phenolics are largely affected by the processing method. Despite some products being fortified with iron, the estimated iron bioavailability among products was very low, with the exception of mycoprotein products and one of the tempeh products. These products did, however, contain a very low amount of iron and, hence, cannot be regarded as good sources of iron.

Bioprocessing, such as fermentation of legumes or production of mycoprotein biomass, has large potential to contribute to the development of nutritious meat substitutes. Such processes, however, require optimization in terms of conditions during fermentation, composition of substrates and choosing appropriate microorganisms and species of filamentous fungus, since these are factors substantially affecting product composition and quality.

Wickramasinghe et al. [77] have previously pointed out significant knowledge gaps in nutritional composition of meat substitutes and highlighted an urgent need for updates to dietary guidelines with regard to real-life dietary patterns of plant-based diets. Our results show that current interpretation of the regulation of nutrition claims of iron can be regarded as misleading, as a very limited amount of the iron present in plant protein products is available for absorption due to the very high content of phytate. Choudhury et al. [78] have previously described imitation of sensory properties of meat as the main driver for formulation of meat substitutes, rather than ensuring an appropriate nutritional composition. Similarly, Tso and Forde [79] raised concern of the risk of an unintentional increase of undesirable nutrients and reduction of overall nutrient density when less-healthy plant-based meat substitutes are selected. Our results are in line with these previous conclusions on meat substitutes.

## 4. Conclusions

Our results point out some nutritional strengths as well as shortcomings of meat substitutes commonly found on the Swedish market. A main area of concern is the very low estimated iron and zinc bioavailability of meat substitutes, caused by the very high phytate content in products based on soy, pea and wheat protein. Mycoprotein products stand out as exceptions when it comes to zinc, and tempeh when it comes to iron. Mycoprotein, however, needs further investigation to evaluate digestibility of the fungal cell walls.

The results in this study highlight the nutritional limitations in terms of iron and zinc bioavailability of shifting from a diet containing animal protein from meat to a diet based on meat substitutes. This study shows difficulties obtaining essential minerals from a diet in which meat has been replaced with products based on legume or cereal proteins, which might lead to an increase in iron deficiency, especially among vulnerable groups. Our results call for a sharpening on the interpretation of nutrition claims, especially for iron, which would create incentive for producers to improve their products with regard to iron bioavailability.

More research is needed to investigate the effects on nutrition and health of extracted and extruded plant proteins.

## Figures and Tables

**Table 1:** Overview of meat substitutes analyzed in this study.

Sample Name	Main Source of Protein	Category
Soy and wheat bacon	Soy and wheat protein 85%	Soy and wheat protein
Soy and wheat balls 1	Soy protein 47%, Wheat protein 14%	Soy and wheat protein
Soy and wheat balls 2	Soy and wheat *	Soy and wheat protein
Soy and wheat balls 3	Soy and wheat protein, 43%	Soy and wheat protein
Soy and wheat burger	Soy protein 58.5%, Wheat protein 13%	Soy and wheat protein
Soy and wheat nuggets	Soy 13%, Wheat 5%	Soy and wheat protein
Soy and wheat sausage	Soy protein, 3.5%; Wheat protein, 10.1%	Soy and wheat protein
Soy and wheat schnitzel	Soy and wheat protein 58%	Soy and wheat protein
Chick pea falafel 1	Chick pea 61%	Whole bean
Chick pea falafel 2	Chick pea, 77%	Whole bean
Farm bean sausage	Farm bean 60%	Whole bean
White bean balls	White beans 51%	Whole bean
Cheese patties 1	Cheese 17%	Cheese
Cheese patties 2	Cheese *	Cheese
Soy balls	Soy protein, 18.5%	Soy protein
Soy burger	Soy protein, 21%	Soy protein
Soy mince 1	Soy protein 29%	Soy protein
Soy mince 2	Soy protein 23%	Soy protein
Soy nuggets	Soy 45%	Soy protein
Soy sausage 1	Soy protein, 17%	Soy protein
Soy sausage 2	Soy protein, 14.9%	Soy protein
Soy schnitzel 1	Soy protein 48%	Soy protein
Soy schnitzel 2	Soy protein, 15%	Soy protein
Pea balls 1	Pea protein *	Pea protein
Pea balls 2	Pea protein *	Pea protein
Pea burger 1	Pea protein *	Pea protein
Pea burger 2	Pea protein 53%	Pea protein
Pea mince	Pea 21%	Pea protein
Pea nuggets	Pea protein *	Pea protein
Pea patties	Pea protein 4%	Pea protein
Pea sausage 1	Pea protein *	Pea protein
Pea sausage 2	Pea protein *	Pea protein
Pea sausage 3	Pea protein, 51%	Pea protein
Pea sausage 4	Pea protein *	Pea protein
Pea schnitzel	Pea protein *	Pea protein
Mycoprotein bites	Mycoprotein 95%	Mycoprotein
Mycoprotein burger	Mycoprotein 40%	Mycoprotein
Mycoprotein Filets	Mycoprotein 85%	Mycoprotein
Mycoprotein mince	Mycoprotein 88%	Mycoprotein
Mycoprotein schnizel	Mycoprotein 38%	Mycoprotein
Oat, pea and bean bites	Oat protein, 17%; Pea protein, 16%, fava bean protein, 16%	Other
Wheat and pea nuggets	Wheat and pea protein *	Other
Wheat fish sticks	Wheat 33%	Other
Tempeh bites	Fermented peas **	Tempeh
Tempeh Burger	Fermented peas **	Tempeh

* Percentage of protein source in the product not available, ** Pure tempeh products.

**Table 2:** Total phenolic content in the analyzed meat substitutes separated and analyzed according to protein source. Data are presented as median value (25th percentile–75th percentile) and are shown as mg gallic acid equivalents (GAE) per 100 g product. ^1^ Results on tempeh are shown as average mg GAE/100 g product ± standard deviation since the sample size was limited to 1.

Group nr	Category	Median
1	Soy and wheat protein, *n* = 8	10.6 (9.6–10.2)
2	Whole bean, *n* = 4	19.3 (7.0–37.7)
3	Cheese, *n* = 2	8.2 (6.9–9.6)
4	Soy protein, *n* = 9	11.7 (9.7–14.9)
5	Pea protein, *n* = 12	10.2 (9.4–26.5)
6	Mycoprotein, *n* = 5	8.5 (7.4–9.8)
7	Other, *n* = 3	8.7 (6.7–14.0)
8	Tempeh ^1^, *n* = 1	21.2 ± 0.9

**Table 3:** Content of total fat, monounsaturated fatty acids (MUFA), polyunsaturated fatty acids (PUFA) and essential fatty acids (FA) in the analyzed meat substitutes, separated and analyzed according to the added fat.

Group Category	Source of Added Fat	Total Fat	Sig.	MUFA	Sig.	PUFA	Sig.	Essential FA ^1^	Sig.
1	No fat (*n* = 3)	2.4 (2.2–2.7)	2, 3 **, 4, 5, 6	0.4 (0.31–0.42)	2, 3, 4, 5	1.00 (0.93–1.32)	2, 3, 4, 5	1.0 (0.93–1.32)	2, 3, 4, 5
2	C, S or P and rapeseed oil (*n* = 5)	16.3 (10.5–17.5)	1, 4, 5	5.76 (3.49–6.76)	1, 3, 4	2.82 (1.53–4.58)	1, 5 **	2.82 (1.52–4.58)	1, 5
3	Rapeseed oil (*n* = 22)	12.8 (9.1–16.2)	1	5.37 (4.06–7.61)	1, 5	3.15 (2.23–4.07)	1, 5 **	3.15 (2.23–4.07)	1, 5 **
4	SF and rapeseed oil (*n* = 6)	10.9 (7.2–12.1)	1, 2	3.99 (2.69–4.69)	1, 2	2.16 (1.63–2.89)	1	2.16 (1.63–2.89)	1, 5 **
5	SF oil (*n* = 7)	13.2 (11.0–14.2)	1, 2	3.15 (2.44–3.21)	1, 2, 3	5.89 (5.65–9.64)	1, 2 **, 3 **	5.89 (5.65–9.64)	1, 2, 3 **, 4 **
6	C and SF oil (*n* = 1)	13.4	1	2.69	-	4.31	-	4.31	-

Data are presented as median g/100 g product (25th percentile–75th percentile). Significance between product categories was analyzed with Mann–Whitney U-test. A *p*-value < 0.05 is considered significant. A *p*-value < 0.01 is illustrated with **, together with the group category number for which the difference was found. Abbreviations: C, coconut oil; S, shea butter; P, palm oil; SF, sunflower oil. ^1^ Essential fatty acids that were present in the meat substitutes were alpha-linolenic acid (ALA) and linoleic acid (LA).

**Table 4:** Salt content and protein content separated and analyzed according to protein source. Data are shown as median value (25th percentile–75th percentile) presented as g/100 g product *n* = number of samples. ^1^ Data on tempeh are presented as average ± measurement uncertainty as reported by Eurofins analytical lab.

Category	Salt Content	Protein Content
Soy and wheat protein, *n* = 8	1.4 (1.1–1.8)	13.0 (11.4–5.4)
Whole bean, *n* = 4	1.3 (1.2–1.4)	8.7 (7.0–11.4)
Cheese, *n* = 2	1.1 (1.1–1.1)	13.3 (11.3–15.3)
Soy protein, *n* = 9	1.1 (1.0–1.2)	12.5 (11.1–14.3)
Pea protein, *n* = 12	1.5 (1.2–2.1)	9.5 (6.9–12.9)
Mycoprotein, *n* = 5	0.7 (0.3–1.0)	13.3 (10.4–14.4)
Other, *n* = 3	1.5 (1.4–1.8)	14.0 (11.9–23.8)
Tempeh ^1^, *n* = 1	1.03 ± 25%	6.7 ± 10%

**Table 5:** Amino acid composition of meat substitutes, sorted into categories based on main protein source (mg/g).

	Soy and Wheat Protein		Soy Protein		Pea Protein		Mycoprotein		Whole Bean		Cheese		Other		Tempeh		mg/kg BW
Essential		(*n* = 8)		(*n* = 9)		(*n* = 12)		(*n* = 5)		(*n* = 4)		(*n* = 2)		(*n* = 3)		(*n* = 1)	per day ^1^
Histidine	3.8	(3.5–4.5)	3.6	(3.1–3.9)	2.8	(2.5–3.4)	3.0	(2.9–3.1)	2.4	(2.3–2.8)	2.6	(2.5–2.7)	3.7	(3.0–3.8)	1.7	± 0.1	10
Isoleucine	6.9	(6.5–7.1)	6.3	(6.0–6.4)	4.9	(4.2–3.4)	6.1	(5.6–6.2)	3.9	(3.7–4.7)	4.7	(4.7–4.8)	6.3	(5.2–6.6)	2.9	± 0.02	20
Leucine	11.9	(11.0–12.0)	10.3	(9.5–10.6)	8.0	(6.8–9.3)	10.2	(8.7–10.7)	6.2	(5.8–8.0)	8.4	(8.1–8.7)	11.8	(9.4–12.1)	3.9	± 0.01	39
Lysine	8.4	(7.6–8.8)	8.1	(7.9–9.1)	7.8	(6.3–8.8)	9.2	(9.0–9.8)	5.2	(84.9–6.6)	6.9	(6.8–7.0)	5.3	(4.0–5.5)	4.3	± 0.05	30
Methionine	1.1	(0.9–1.8)	1.1	(1.0–1.6)	1.0	(0.8–1.3)	2.3	(2.0–2.4)	1.1	(1.0–1.3)	1.9	(1.7–2.1)	1.5	(1.3–1.5)	1.2	± 0.02	
Phenylalanine	8.3	(7.8–8.4)	7.0	(6.9–7.3)	5.6	(4.6–6.3)	6.1	(5.2–6.2)	4.4	(4.2–5.3)	5.2	(5.1–5.4)	8.7	(6.6–8.8)	2.9	± 0.04	25 ^2^
Threonine	6.2	(6.0–6.4)	5.8	(5.2–5.9)	4.3	(3.7–4.9)	6.1	(5.9–6.3)	3.5	(3.2–4.5)	4.0	(3.9–4.1)	4.9	(4.3–5.3)	2.8	± 0.00	15
Valine	7.1	(6.6–7.4)	6.3	(6.0–6.3)	5.0	(4.5–5.8)	6.7	(6.6–7.0)	4.2	(3.9–5.2)	5.7	(5.6–5.9)	6.7	(5.4–7.0)	3.1	± 0.00	26
Non essential																	
Alanine	6.4	(5.9–6.6)	5.7	(5.3–5.8)	4.5	(4.0–5.2)	6.7	(6.4–6.9)	3.9	(3.6–4.7)	3.8	(3.7–3.9)	4.7	(4.2–5.1)	3.0	± 0.1	-
Arginine	8.5	(6.8–8.9)	8.6	(6.4–9.4)	6.9	(6.1–8.3)	6.2	(5.7–6.8)	5.3	(4.9–7.0)	3.6	(3.4–3.7)	6.2	(5.5–6.7)	2.7	± 0.1	
Aspartic acid	14.3	(12.7–14.8)	13.8	(13.6–15.4)	11.5	(9.6–13.1)	10.9	(10.8–11.3)	8.9	(8.2–11.0)	7.5	(7.4–7.6)	9.8	(8.3–9.8)	6.0	± 0.02	-
Cysteine	0.5	(0.1–1.0)	0.2	(0.1–0.5)	0.4	(0.3–0.5)	0.4	(0.4–0.5)	0.05	(0.05–0.05)			0.4	(0.3–0.5)			-
Glutamic acid	34.1	(32.2–36.2)	25.7	(22.3–27.1)	16.6	(13.9–20.0)	15.8	(13.8–20.5)	12.5	(12.4–15.7)	18.8	(18.0–19.6)	48.3	(31.9–54.1)	7.9	± 0.1	-
Glycine	5.9	(5.5–6.3)	5.3	(4.6–5.5)	4.1	(3.5–4.7)	4.7	(4.6–4.8)	3.4	(3.3–4.1)	2.6	(2.6–2.7)	5.5	(4.5–5.7)	2.4	± 0.2	-
Proline	10.7	(10.0–11.3)	7.2	(6.7–8.0)	4.6	(3.9–5.4)	5.7	(4.9–8.3)	3.7	(3.7–4.5)	8.3	(7.9–8.7)	16.8	(10.5–18.8)	2.5	± 0.04	-
Serine	8.2	(7.7–8.7)	7.1	(6.5–7.4)	5.4	(4.8–6.2)	6.8	(6.0–6.8)	4.6	(4.4–5.8)	5.7	(5.6–5.8)	8.5	(6.6–8.7)	3.1	± 0.06	-
Tyrosine	6.0	(5.8–6.3)	5.4	(5.3–5.7)	4.4	(4.0–4.9)	5.0	(4.4–5.1)	3.7	(3.6–4.3)	4.8	(4.7–4.9)	6.1	(4.7–6.2)	2.4	± 0.00	-

Data presented as median (25th percentile–75th percentile), mg/g product. Data on tempeh is presented as average out of duplicates standard deviation. ^1^ Amino acid recommendations. Calculated as mg/kg body weight for adults based on a total protein requirement of 0.66 g/kg BW per day, according to WHO/FAO/UNU 2007. ^2^ Phenylalanine and tyrosine combined.

**Table 6:** Overview of total dietary fiber and the soluble and insoluble fractions of dietary fibers in meat substitutes, categorized according to the main protein source.

Meat Substitutes Based on:	Total DF	Soluble DF	Insoluble DF
Soy and wheat protein extract, *n* = 8	10.9 (8.0–13.9)	3.3 (4.5–4.9)	7.7 (5.9–10.0)
Whole bean, *n* = 4	11.4 (9.2–13.2)	3.6 (2.1–5.1)	6.8 (6.6–10.0)
Soy protein extract, *n* = 9	10.5 (9.6–13.3)	4.3 (3.3–4.9)	6.7 (6.0–8.2)
Pea protein extract, *n* = 12	8.0 (7.1–9.7)	2.7 (2.1–3.3)	5.3 (4.3–6.6)
Mycoprotein, *n* = 5	9.9 (6.8–17.8)	1.3 (1.0–3.4)	7.4 (5.8–16)
Tempeh, *n* = 1	21.5	6.1	15.4
Cheese, *n* = 2	6.7 (6.2–7.3)	2.2 (1.8–2.5)	4.6 (4.4–4.8)
Other, *n* = 3	5.6 (4.4–8.8)	1.4 (0.5–4.3)	4.1 (3.9–4.5)

Dietary fiber (DF) and fiber fractions are presented as percentage of total dry weight. Results are presented as median (25th–75th percentile). Data on tempeh are presented as average value from duplicates.

## Data Availability

Data described in the manuscript, code book, and analytic code will be made available upon request pending approval.

## Supplementary Materials

The following supporting information can be downloaded at: https://www.mdpi.com/article/10.3390/nu14193903/s1, Table S1: Added fat in analyzed meat substitutes.
