# Supplementary material for: Nutritional Composition and Estimated Iron and Zinc Bioavailability of Meat Substitutes Available on the Swedish Market
Source: Nutrients. 2022 Sep 21;14(19):3903. doi: 10.3390/nu14193903 (PMC9571894; doi:10.3390/nu14193903)
Supplement: Supplementary file 1 [file nutrients-14-03903-s001.zip › nutrients-1897524-supplementary.pdf]

**Table S1. Source of added fat, as stated on the packaging of the product, for each meat substitute included in the study**

| Product name | Added fat |
|---|---|
| Cheese patties 1 | Sunflower oil |
| Cheese patties 2 | Sunflower and rapeseed oil |
| Chick pea falafel 1 | Rapeseed oil |
| Chick pea falafel 2 | Rapeseed oil |
| Farm bean sausage | Sunflower oil |
| Mycoprotein bites | No added fat |
| Mycoprotein burger | Coconut, shea or palm and rapeseed |
| Mycoprotein filets | No added fat |
| Mycoprotein mince | No added fat |
| Mycoprotein schnizel | Sunflower and rapeseed oil |
| Oat and bean bites | Rapeseed oil |
| Pea balls 1 | Rapeseed oil |
| Pea balls 2 | Rapeseed oil |
| Pea burger 1 | Coconut, shea or palm and rapeseed |
| Pea burger 2 | Coconut and sunflower oil |
| Pea mince | Rapeseed oil |
| Pea nuggets 1 | Rapeseed oil |
| Pea patties | Rapeseed oil |
| Pea sausage 1 | Coconut, shea or palm and rapeseed |
| Pea sausage 2 | Coconut, shea or palm and rapeseed |
| Pea sausage 3 | Sunflower oil |
| Pea sausage 4 | Rapeseed oil |
| Pea schnitzel | Rapeseed oil |
| Soy and wheat bacon | Sunflower oil |
| Soy and wheat balls 1 | Sunflower and rapeseed oil |
| Soy and wheat balls 2 | Rapeseed oil |
| Soy and wheat balls 3 | Sunflower oil |
| Soy and wheat burger | Sunflower and rapeseed oil |
| Soy and wheat nuggets | Rapeseed oil |
| Soy and wheat sausage | Sunflower and rapeseed oil |
| Soy and wheat schnitzel | Sunflower oil |
| Soy balls | Rapeseed oil |
| Soy burger | Rapeseed oil |
| Soy mince 1 | Rapeseed oil |
| Soy mince 2 | Rapeseed oil |
| Soy nuggets | Sunflower and rapeseed oil |
| Soy sausage 1 | Rapeseed oil |
| Soy sausage 2 | Coconut, shea or palm and rapeseed |
| Soy schnitzel 2 | Sunflower oil |
| Soy schnitzel 3 | Rapeseed oil |
| Tempeh Burger | Rapeseed oil |
| Wheat and pea nuggets | Rapeseed oil |
| Wheat fish sticks | Rapeseed oil |
| White bean balls | Rapeseed oil |
